# Some Experiences of Training-School Life

**Published:** 1918-08-03

**Authors:** 


### THE MATRONS' AND SISTERS' DEPARTMENT.

### SOME EXPERIENCES OF TRAINING-SCHOOL LIFE.

### II. Some Hardening Influences in Training.

It is not perhaps always sufficiently recognised in hospitals that the first few days may be the making or the marring of a nurse. Not, of course, in every case, for the diversity of nature among probationers is infinite. But most new probationers, if at all sympathetic, have an instinctive desire to respond in some way to every request made by the patients, and the carelessly flung and often harshly worded remarks of the senior—at the patients' expense—are not calculated, however much truth there may be in them, to foster gentleness in the probationer; the latter may quite possibly receive the impression that this is the correct attitude, and that to look at things from the patient's point of view is a sign of weakness and inefficiency. Nor is that absorption in the business in hand which prevents a staff nurse (when demonstrating treatment to a junior) from seeing—or if she does see, from noticing—the natural shrinking of her patient, a good education in true sympathy.

The necessary and excellent discipline of a large hospital decrees that a fixed routine must be observed. However gentle her instincts may be, the nurse knows that, for example, so many dressings have got to be finished within a given time if the whole routine of the ward is not to be upset. Her mind, therefore, is frequently on the clock and not on her patient, and in the race against time she may exhibit a roughness and callousness quite foreign to her nature, but which, if the pace has to be sustained week after week, becomes an unconscious habit. Many nurses can, in hospital slang, "do a sprint" on a special occasion, and manage at the same time to show consideration, or at the least, by means of a light-hearted badinage, carry their patients with them and make all contented. But if wards are continuously heavy, off-duty time inadequate, and holidays long overdue, the gaiety becomes a physical impossibility, and is replaced by impatience and irritability either vented or else held in too obvious check—with resulting unhappiness to the patient, and too frequently a permanent hardness in the nurse.

In the early days of the war our senses were wont to reel at the appalling list of casualties, or at the figures involved in the carrying on of the war. Now our lips toss millions lightly about, and our imaginations are inured to mental pictures of the direst catastrophes. So may it be with the nurse. Her life is spent in the midst of suffering of all degrees; it is her normal entourage. Familiarity with it breeds not contempt, but unconscious indifference. Sights that at first caused her almost to faint become a commonplace of everyday life. Pain and suffering endured by the patients, so intense as to stir the depths of her soul with pity, becomes daily less intense in her consciousness because it is always there. The sacredness of death, the instinctive hush that comes to the spirit in the presence of the great unknown, are dulled by frequent contact, or replaced by anxious haste to get the resulting work done, and an inevitable consideration of the extra time needed and the interference with routine. The nurse may not realise it herself, and certainly has no conception of the impression it makes upon other people. Happily this is by no means a universal occurrence, and in many nurses their reverence for the great mysteries of life is increased rather than diminished by their training. But where there is a natural lack of imagination, indifference results from familiarity far more surely than where the imagination is keen and the spiritual consciousness fully developed.

More deeply founded and harder to deal with than any of these, in that it affects often the very best and gentlest of nurses, is the hardness of self-defence. Many a sensitive nurse, who feels the pain and suffering of others as keenly as if it were her own, finds herself obliged to assume a hardness which she does not feel if she is to do her work successfully. She realises that more practical good is accomplished for the patient by the doing of things than by the outward expression of sympathy, and partly as a reaction against the helplessness of mere words, partly as a protection against her own sensitive feelings, there gradually forms round her a kind of outer-crust or shell which is proof against the darts of everyday hospital occurrences.

A hospital is not merely a collection of sick folk. It is a small world, embracing within its walls many sorts and conditions of men and women, and quite a variety of interests. The probationer has many things to think of besides her patients—things altogether new to her, and frequently so difficult from her point of view that they occupy a disproportionately large place in her consciousness. Hospital etiquette is in itself a distinct branch of learning, and woe awaits the breach of its strict rules. Many things there are to be understood which apparently have nothing to do with the patients or the nursing of them, and the keener the probationer the more attention she bestows upon these things, and the more anxious she is about their precise observance. Then comes the theoretical side of her work, the lectures and classes, presenting new and baffling problems, besides being a source of intense interest demanding a great deal of concentration. All this is excellent training and discipline in itself, but tends to develop the defects of the qualities it is meant to produce, so that the appearance of the ward becomes a consideration more important than the comfort of the patients, a quickened intellectual interest in "the case" is accompanied by a diminished sympathetic interest in the human being, and self-reliance and coolness are carried to the point of callousness.

☛ The previous article appeared July 20, p. 349.

